# Potential diagnostic and prognostic values of detecting promoter hypermethylation in the serum of patients with gastric cancer

**DOI:** 10.1038/sj.bjc.6602636

**Published:** 2005-06-07

**Authors:** W K Leung, K-F To, E S H Chu, M W Y Chan, A H C Bai, E K W Ng, F K L Chan, J J Y Sung

**Affiliations:** 1 Department of Medicine & Therapeutics, The Chinese University of Hong Kong, Prince of Wales Hospital, 30-32 Ngan Sing Street, Shatin, Hong Kong; 2 Department of Anatomical & Cellular Pathology, The Chinese University of Hong Kong, Prince of Wales Hospital, Shatin, Hong Kong; 3 Department of Surgery, The Chinese University of Hong Kong, Prince of Wales Hospital, Shatin, Hong Kong

**Keywords:** gastric cancer, promoter hypermethylation, tumour suppressor genes, oncogenes

## Abstract

While there is no reliable serum biomarker for the diagnosis and monitoring of patients with gastric cancer, we tested the potential diagnostic and prognostic values of detecting methylation changes in the serum of gastric cancer patients. DNA was extracted from the pretherapeutic serum of 60 patients with confirmed gastric adenocarcinoma and 22 age-matched noncancer controls. Promoter hypermethylation in 10 tumour-related genes (*APC*, *E-cadherin*, *GSTP1*, *hMLH1*, *MGMT*, *p15*, *p16*, *SOCS1*, *TIMP3* and *TGF-beta RII*) was determined by quantitative methylation-specific PCR (MethyLight). Preferential methylation in the serum DNA of gastric cancer patients was noted in *APC* (17%), *E-cadherin* (13%), *hMLH1* (41%) and *TIMP3* (17%) genes. Moreover, patients with stages III/IV diseases tended to have higher concentrations of methylated *APC* (*P*=0.08), *TIMP3* (*P*=0.005) and *hMLH1* (*P*=0.03) in the serum. In all, 33 cancers (55%) had methylation detected in the serum in at least one of these four markers, while three normal subjects had methylation detected in the serum (specificity 86%). The combined use of *APC* and *E-cadherin* methylation markers identified a subgroup of cancer patients with worse prognosis (median survival 3.3 *vs* 16.1 months, *P*=0.006). These results suggest that the detection of DNA methylation in the serum may carry both diagnostic and therapeutic values in gastric cancer patients.

Gastric cancer is the second most common cause of cancer-related mortality in the world that killed more than 640 000 patients each year (Globocan, 2000). Although screening for gastric cancer may improve the overall survival of cancer patients by identifying early cancers, a reliable simple and noninvasive screening test is lacking. In particular, there is no serum biomarker for gastric cancer. The recent rapid advancement in molecular or biochemical techniques may help to identify novel serum markers to be used for this purpose. Apart from screening, these serum biomarkers may also help to stratify cancer patients according to the risk of recurrence.

Epigenetic silencing of tumour-associated genes by promoter hypermethylation is increasingly recognised to play an instrumental role in cancer development (Egger *et al*, 2004). These changes, which involve DNA and histone modifications, result in the heritable silencing of genes without a change in their coding sequence. We and others have previously demonstrated the frequent hypermethylation of CpG islands within the promoter regions of genes like *hMLH1*, *E-cadherin*, *p15*, *p16*, and *APC* in gastric cancer (Tsuchiya *et al*, 2000; Leung *et al*, 2001; To *et al*, 2002; Kang *et al*, 2003; Sarbia *et al*, 2004). Moreover, these epigenetic alterations could be readily detected in the tumour-derived DNA recovered from the serum of gastric cancer patients (Lee *et al*, 2002). With the growing number of methylation markers and the development of high-throughput techniques, we determined the potential diagnostic and prognostic significance of detecting gene methylation in the serum DNA of patients with adenocarcinoma of stomach by the use of a quantitative DNA methylation assay, MethyLight.

## MATERIALS AND METHODS

### Patients and control

A total of 60 Chinese patients with confirmed gastric adenocarcinoma were examined (male 58.3%, mean age=66 years, range 35–96 years). The majority (78%) of cancers were located in the distal stomach. None of these patients had family history of gastric cancer. In all, 22 age- and gender-matched subjects with normal upper gastroscopy were included as control. All blood samples were collected at the time of diagnosis, usually at the time of endoscopy, prior to any therapeutic intervention. Baseline demographic data of patients were recorded. Tumour was staged according to the sixth edition of the TNM staging system (Sobin and Wittekind, 2002). Staging information was available in 54 patients. There were five stage I, seven stage II, 13 stage III and 29 stage IV cancers. All cancer patients were treated according to a standard protocol with surgery being the mainstay of treatment. Patients were being regularly followed up in our clinic and the median follow-up duration since the time of diagnosis was 8 months (range 0– to 40 months). In total, 34 (56.7%) patients died in the follow-up period. Two patients died in the early postoperative period were excluded in the subsequent survival analysis.

All patients and controls gave informed consent for participation in this study and the study protocol was approved by the Clinical Research Ethics Committee of the Chinese University of Hong Kong.

### DNA extraction and modification

Serum samples obtained from cancer patients and controls were randomly coded before processing to ensure adequate blinding of the clinical information. The serum was separated by centrifugation and stored at −20°C prior to processing. Genomic DNA was extracted from 800 *μ*l serum with commercially available DNA extraction kit (QIAamp Blood Kit; Qiagen Hilden, Germany). The DNA was then chemically modified by sodium bisulphite to convert all unmethylated cytosines to uracils while leaving methylcytosines unaltered (EZ DNA methylation kit; Zymo Research, Orange, CA, USA), and eluted in 50 *μ*l of elution buffer.

### Methylation-specific PCR (MSP)

The fluorescence-based real-time PCR assay, MethyLight, was used in the detection of methylated DNA in the serum (Eads *et al*, 2000, 2001). A total of 10 tumour-related genes were examined: *APC*, *E-cadherin*, *GSTP1*, *hMLH1*, *MGMT*, *p15*, *p16*, *SOCS1*, *TIMP3* and *TGF-beta RII*. These genes were previously reported to be methylated in various human cancers (Eads *et al*, 2001; To *et al*, 2004). The sequences of the primers and fluorogenic probes were listed in Table 1.

For each amplification, 5 *μ*l of bisulphite converted DNA, equivalent to DNA extracted from 80 *μ*l of the serum, was used. PCR was performed in a 25 *μ*l reaction volume consisting of 5 pmol of each primer, 250 pmol of probe, 200 *μ*M each of dATP, dCTP and dGTP, 400 *μ*M dUTP, 3.5mM MgCl_2_, 1 × TaqMan Buffer A and 2 U of AmpliTaq Gold polymerase at the following condition: 95°C for 10 min, followed by 50 cycles at 95°C for 15 s and 60°C for 1 min. All PCR was performed in iCycler iQ Real-Time PCR Detection system (BioRad, Hercules, CA, USA). CpGenome™ Universal methylated DNA (Chemicon International Inc., CA, USA) was included in all amplifications as positive control and internal reference, whereas bisulphite-modified human sperm DNA was used as negative control. A standard curve was created by plotting the logarithmic of the amount of standard universal methylated DNA in the range of 31.25 pg–10 ng against the threshold cycle value. The minimal correlation coefficient for each quantitative MSP was 0.98. The corresponding amount of methylated DNA in the serum samples was calculated from the standard curve. A serum DNA sample was considered to be methylated when the level of methylated DNA was greater than or equivalent to 0.05 ng in 80 *μ*l serum. This level was determined after adjustment to the background level detected in normal subjects (data not shown).

A methylation marker was considered to be preferentially methylated in gastric cancer patients when the following criteria were met (Muller *et al*, 2003): (1) unmethylated in serum samples of healthy control but methylated in more than 10% of serum samples from gastric cancer patients or (2) ⩽10% methylated in serum samples of normal control but methylated in >20% of serum samples of gastric cancer patients.

### Statistical analysis

Statistical analysis was performed by using SPSS software (version 11.5; SPPS, Chicago, IL, USA). Categorical data were analysed by Fisher's exact test, whereas numerical value was compared by Student's *t*-test. One-way ANOVA was used in the comparison of the concentrations of methylated DNA among control subjects, and patients with early (stages I and II) and advanced (stages III and IV) cancer. The Kaplan–Meier method was used for univariate survival analysis and the log-rank test was used to compare the difference in survival curves. The presence of methylated DNA in the serum was analysed in a dichotomous manner (i.e. methylated or unmethylated) during survival analysis. Those who died from postoperative complications were excluded for survival analysis. A *P*-value of less than 0.05 was considered to be statistically significant.

## RESULTS

### DNA methylation in the serum

**Figure 1:**
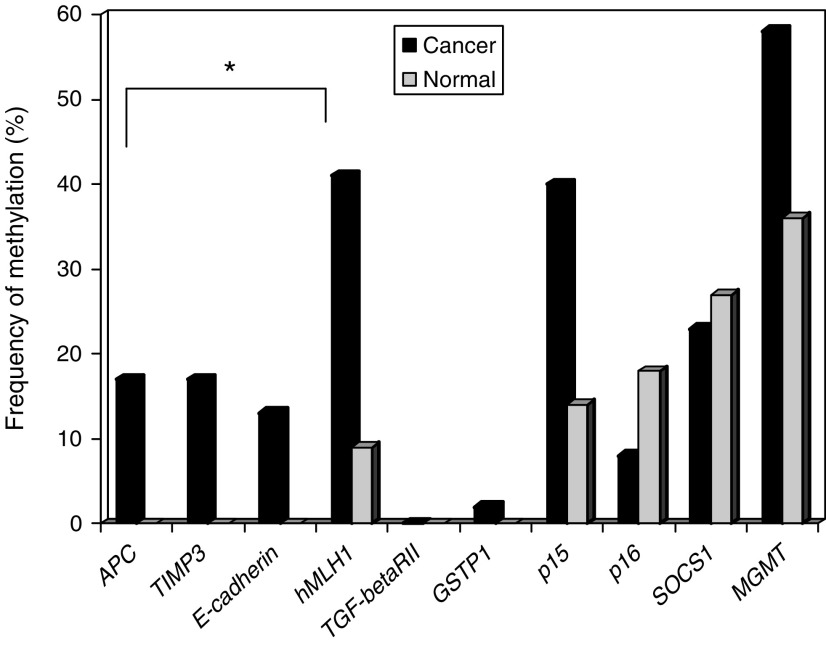
Frequency of detecting methylated DNA in the serum of gastric cancer patients and control. ^*^Genes with differential methylation in cancer patients according to predefined criteria.

The frequency of DNA methylation in the serum of gastric cancer patients and controls was shown in Figure 1. By using the predefined criteria (Muller *et al*, 2003), differential methylation was detected in *APC*, *E-cadherin*, *hMLH1* and *TIMP3*. The frequency of detecting methylated DNA in the serum of cancer patients for *APC* was 17%, *E-cadherin* was 13%, *hMLH1* was 41% and *TIMP3* was 17%. On the other hand, differential methylation pattern was not observed in the remaining tumour-related genes due to either lack of specificity (p15, p16, SOCS1 and MGMT) or low methylation frequency (*TGF-beta RII* and *GSTP1*).

To further test the specificity of detecting methylated DNA in serum, we determined the methylation status of *APC*, *E-cadherin*, *hMLH1* and *TIMP3* in primary gastric cancer tissues by MSP. Among the 33 gastric cancers with DNA available for analysis, the corresponding number of cases with promoter hypermethylation in primary cancer tissues for *APC*, *E-cadherin*, *hMLH1* and *TIMP3* was 20 (61%), five (15%), 14 (42%) and five (15%). It was apparent from this paired comparison that nearly all tumours with methylation in *E-cadherin*, *hMLH1* and *TIMP3* had methylation detected in the serum DNA as well. Moreover, all patients with methylation detected in the serum DNA had methylation in the corresponding tumours.

**Figure 2:**
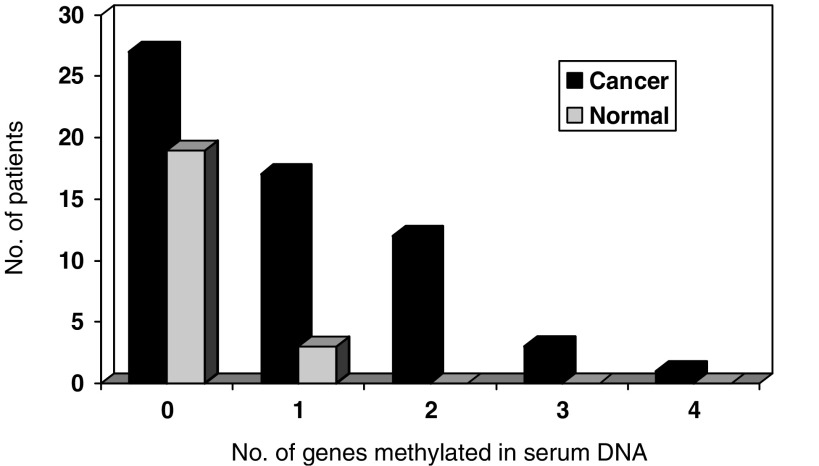
Number of genes methylated in the serum of gastric cancer patients and control.

With the combined use of the four methylation markers that exhibited differential methylation in cancer (*APC*, *E-cadherin*, *hMLH1* and *TIMP3*), 33 (55%) patients had methylated serum DNA detected in at least one of these markers. In contrast, three normal subjects had methylated DNA detected in the serum, and hence, the specificity of this panel of markers was 86%. With the use of this panel of markers, the median number of genes methylated in the serum of cancer patients and control was 1 and 0, respectively (Figure 2).

### Clinicopathological correlation

**Figure 3:**
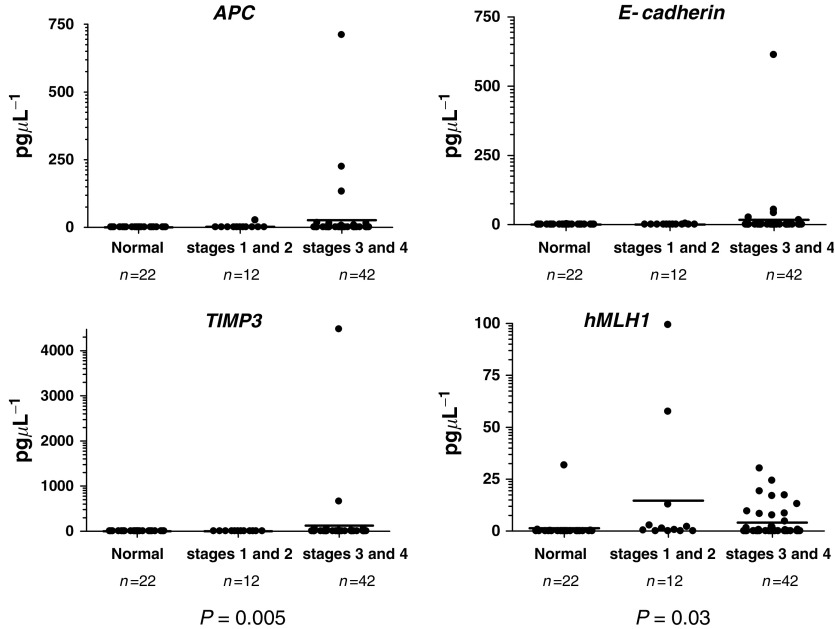
Association between cancer staging and concentrations of methylated DNA. (**A**) *APC* (*P*=0.08); (**B**) *E-cadherin* (*P*=0.2); (**C**) *TIMP3* (*P*=0.005) and (**D**) *hMLH1* (*P*=0.03). The horizontal line indicated the median concentrations of the patients’ subgroups.

High level of methylated DNA was more frequently detected in the serum of patients with more advanced cancer (Figure 3). Specifically, patients with stages III/IV diseases tended to have higher concentrations of methylated *APC* (*P*=0.08), *TIMP3* (*P*=0.005) and *hMLH1* in the serum (*P*=0.03). On the other hand, there was no significant association between the levels of methylated *E-cadherin* DNA and tumour staging (*P*=0.2).

The potential association between methylated DNA in serum and patients’ demographic data was investigated. Notably, patients with methylated *hMLH1* in the serum were slightly younger than patients with unmethylated *hMLH1* (61.2 *vs* 69.3 years, *P*=0.045). There was no other association between the presence of methylated DNA in the serum and patients’ characteristics.

### DNA methylation and survival

**Figure 4:**
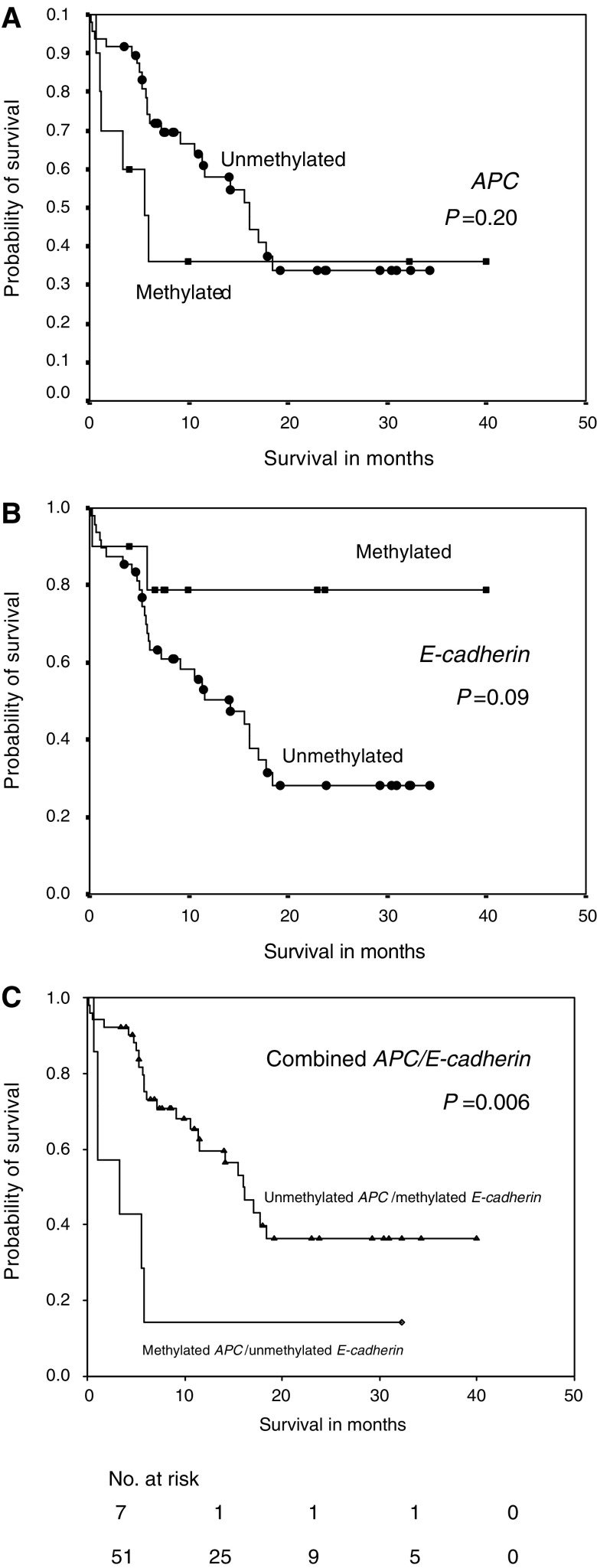
Kaplan–Meier analysis of the probability of overall survival in gastric cancer patients according to methylation status. (**A**) *APC* (*P*=0.20); (**B**) *E-cadherin* (*P*=0.09); (**C**) combined *APC* and *E-cadherin* (*P*=0.006).

Apart from determining its diagnostic accuracy, we examined the potential prognostic value of detecting DNA methylation in the pretherapeutic serum of gastric cancer patients. Patients with methylated *APC* DNA in serum tended to have a nonsignificant shorter survival than patients with unmethylated *APC*. The difference appeared to be more marked in the initial 18 months of diagnosis. The overall median survival of patients with and without methylated *APC* in serum was 5.5 and 16.1 months, respectively (*P*=0.20, Figure 4A). Conversely, those with methylated *E-cadherin* tended to have a longer survival than patients with unmethylated gene (median survival 32.1 *vs* 14.1 months, *P*=0.09, Figure 4B). The presence of methylated *hMLH1* and *TIMP3* DNA in the serum was not associated with overall patients’ survival (*P*=0.40 and 0.58, respectively).

When combining the two methylation markers *APC* and *E-cadherin*, patients with methylated *APC* and unmethylated *E-cadherin* in the serum were found to have a significantly worse prognosis when compared to those without these alterations (median survival 3.3 *vs* 16.1 months, *P*=0.006, Figure 4). Other combinations of methylation markers were not found to have any correlations with patients’ survival.

## DISCUSSION

Unlike many other cancers, a reliable serum biomarker for gastric cancer is lacking. In particular, a serum marker that may carry both diagnostic and prognostic implication is currently unavailable. In this study, we characterised the significance of detecting DNA methylation in the serum of gastric cancer patients. Of the 10 methylation markers examined, differential methylation was noted in the following genes: *APC*, *E-cadherin*, *hMLH1* and *TIMP3*. By using this panel of methylation markers, 55% of gastric cancer had methylated DNA detected in the serum. This is higher than the previous report by Koike *et al* (2004) in which 44% of patients were found to have methylation in *p16*, *E-cadherin* and/or *RARβ*.

Furthermore, we determined the concentration of methylated DNA in the serum of cancer patients and controls by using the quantitative MSP assay. We found that the concentrations of methylated *APC*, *hMLH1* and *TIMP3* were higher in patients with advanced stage cancer. Similar stage-dependent increase in methylated *APC* DNA was also noted in patients with oesophageal adenocarcinoma (Kawakami *et al*, 2000), which may be related to the heavy tumour load in patients with more advanced cancer. Although Nakajima *et al* (2001) showed that frequency of *hMLH1* methylation in gastric cancer significantly increase with ages, we found that the detection of methylated *hMLH1* in the serum was slightly more common in younger patients. Further studies may be needed to characterise the sources and mechanisms of tumour DNA circulating in blood to give a better explanation for this observation.

In this study, we found that the presence of methylated *APC* or unmethylated *E-cadherin* in the pretherapeutic serum of cancer patients tended to have a nonsignificant trend towards poor survival. In particular, the presence of methylated *APC* and unmethylated *E-cadherin* in the serum had a lower survival chance than those without these alterations. Recently, several studies have demonstrated the potential prognostic significance of detecting aberrant promoter hypermethylation in the serum/plasma of patients with oesophageal (Kawakami *et al*, 2000), lung (Usadel *et al*, 2002) and breast (Muller *et al*, 2003) cancers. Kawakami *et al* (2000) reported that the presence of high plasma levels of methylated *APC* DNA were associated with reduced survival in patients with oesophageal adenocarcinoma. In addition, Muller *et al* (2003) demonstrated that the presence of methylated *APC* and/or *RASSF1A* was associated with poor outcome in patients with breast cancer. Interestingly, our data showed that patients with methylated *E-cadherin* in serum tended to have a higher survival chance. Past studies found that *E-cadherin* methylation may be more common in the undifferentiated diffuse-type gastric cancer (Tamura *et al*, 2000). On the other hand, there was no reported association between the presence of *E-cadherin* methylation in the serum and survival of cancer patients. It remains elusive whether the difference in survival is related to the preferential methylation in *E-cadherin* in different histological subtypes of cancer. Owing to the relatively small sample size and the low frequency of *E-cadherin* in the serum, we were unable to perform subgroup analysis according to histological subtypes. Studies with larger sample size may be necessary to discern the potential prognostic significance of detecting *E-cadherin* methylation in the serum of gastric cancer patients with different histological subtypes.

Based on the differential methylation patterns of different methylation markers, it is obvious that the sensitivity and specificity of these methylation markers may vary in different tumour types. As shown by previous studies (Eads *et al*, 2001; Muller *et al*, 2003), some of these methylation markers are detected in very high frequency even in the serum of normal individuals, whereas some markers are never methylated in the serum of cancer patients. By using the predefined criteria, we have determined the panel of markers that are specific for gastric cancer. The detection rates of this study were different from our previous report (Lee *et al*, 2002), which may be accounted by the changes in methylation detection methods. In particular, methylated *E-cadherin* was detected in the serum of more than 50% of cancer patients by using conventional MSP in our previous study. By using quantitative MethyLight assay, a lower detection rare of 13% was obtained. Apart from the difference in PCR primers, the MethyLight includes an internal fluorogenic probe, which increases the specificity of the assay.

The major advantage in detecting aberrantly methylated DNA in the serum is the convenience and simplicity of the test. With the use of high-throughput fluorescence-based real-time PCR (MethyLight), multiple tumour-related genes or multiple patients’ samples can be processed at the same time. For instance, a typical single PCR reaction takes less than 2 h and could provide invaluable information on screening as well as on prognosis. With the identification of more and more methylation markers, it is anticipated that the positive rates, or sensitivity, of detecting methylated DNA in the serum of gastric cancer patients will rise. It will also be interesting to determine in future studies whether aberrantly methylated serum DNA would be useful in monitoring of disease progression or treatment response in patients with gastric cancer.

## Figures and Tables

**Table 1:** Summary of primer sequences and probe used for quantitative MSP

**Marker**	**Primer sequences**	**Probe**	**Ref.**
*APC*	GAACCAAAACGCTCCCCAT (forward) TTATATGTCGGTTACGTGCGTTTATAT (reverse)	6FAM-CCCGTCGAAAACCCGCCGATTA-TAMRA	Eads *et al*, 2001
*E-cadherin*	AATTTTAGGTTAGAGGGTTATCGCGT (forward) TCCCCAAAACGAAACTAACGAC (reverse)	6FAM-CGCCCACCCGACCTCGCAT-TAMRA	Eads *et al*, 2001
*GSTP1*	GTCGGCGTCGTGATTTAGTATTG (forward) AAACTACGACGACGAAACTCCAA (reverse)	6FAM-AAACCTCGCGACCTCCGAACCTTATAAAA-TAMRA	Eads *et al*, 2001
*hMLH1*	CGTTATATATCGTTCGTAGTATTCGTGTTT (forward) CTATCGCCGCCTCATCGT (reverse)	6FAM-CGCGACGTCAAACGCCACTACG-TAMRA	Eads *et al*, 2001
*p15*	AGGAAGGAGAGAGTGCGTCG (forward) CGAATAATCCACCGTTAACCG (reverse)	6FAM-TTAACGACACTCTTCCCTTCTTTCCCACG-TAMRA	Eads *et al*, 2001
*p16*	TGGAATTTTCGGTTGATTGGTT (forward) ACAACGTCCGCACCTCCT (reverse)	6FAM-ACCCGACCCCGAACCGCG-TAMRA	Eads *et al*, 2001
*TIMP3*	GCGTCGGAGGTTAAGGTTGTT (forward) CTCTCCAAAATTACCGTACGCG (reverse)	6FAM-AACTCGCTCGCCCGCGAA-TAMRA	Eads *et al*, 2001
*MGMT*	CTAACGTATAACGAAAATCGTACAACC (forward) AGTATGAAGGGTAGGAAGAATTCGG (reverse)	6FAM-CCTTACCTCTAAATACCAACCCCAAACCCG-TAMRA	Eads *et al*, 2001
*TGF-beta RII*	GCGCGGAGCGTAGTTAGG (forward) CAAACCCCGCTACTCGTCAT (reverse)	6FAM-CACGAACGACGCCTTCCCGAA-TAMRA	Eads *et al*, 2001
*SOSC1*	ACGTCGATTATCGGCGTATTAC (forward) CGCTCAAAAACCCCCAAT (reverse)	6FAM-TTTGGACGTTTGCGGATTT-TAMRA	To *et al*, 2004

MSP=methylation-specific PCR.
